# Triple‐energy photon‐counting x‐ray imaging for bone‐strontium estimation: A simulation study

**DOI:** 10.1002/mp.18125

**Published:** 2025-09-23

**Authors:** Jesse Tanguay, Bobby Tang, Eric Da Silva

**Affiliations:** ^1^ Department of Physics Toronto Metropolitan University Toronto Canada

**Keywords:** bone strontium quantification, photon‐counting x‐ray imaging, quantitative imaging, spectral imaging, triple‐energy imaging

## Abstract

**Background:**

Strontium quantification in bone is clinically relevant but typically requires specialized stand‐alone systems. Photon‐counting detectors offer energy‐resolved imaging that may enable low‐dose estimation of both strontium concentration and bone mineral density in a single acquisition.

**Purpose:**

To evaluate the feasibility of triple‐energy photon‐counting x‐ray imaging for low‐dose quantification of strontium in bone, using a simulation framework that accounts for energy bin sensitivity, detector noise, and anatomical geometry.

**Methods:**

A forward model of a photon‐counting detector was used to simulate energy‐resolved x‐ray measurements through a simplified model of the human finger, incorporating cortical bone, trabecular bone, and soft tissue. Strontium uptake was modeled as a mass concentration relative to bone. A generalized least‐squares estimator was used to compute the strontium‐to‐bone concentration from energy‐resolved measurements. We optimized tube voltage and energy thresholds for three clinically relevant anode/filter combinations and three levels of electronic noise (5, 10, and 15 keV), with the goal of minimizing the limit of quantification (LOQ) and absorbed dose. A Fisher information analysis was conducted to assess the relative contribution of each energy bin to estimation precision.

**Results:**

Optimal tube voltages and thresholds depended strongly on electronic noise but only modestly on anode/filter choice. At a 5 keV noise floor, an LOQ of 100 ppm could be achieved with an absorbed dose of ∼13 μGy, whereas 10 and 15 keV noise levels required ∼100 μGy and >175 μGy, respectively. At a fixed dose of 20 μGy, reliable detection (SNR > 1) was possible at concentrations as low as 50 ppm for 5  and 10 keV noise floors. The mid‐energy bin consistently contributed the most to estimation precision across all scenarios. At low noise, the high‐energy bin was second most informative; at higher noise levels, the low‐energy bin overtook it due to shifting energy thresholds that placed the strontium K‐edge (∼16 keV) in the lower bins.

**Conclusions:**

Triple‐energy photon‐counting x‐ray imaging offers a promising strategy for low‐dose quantification of strontium in bone. Its performance is primarily limited by electronic noise, while spectral shaping through anode and filter selection plays a secondary role when acquisition parameters are optimized.

## 1 INTRODUCTION

Strontium (Z=38) is a naturally occurring element in bone, and presents remarkably similar chemistry to calcium as it is an alkali Earth metal.^1^ It can have either beneficial or adverse effects on bone health depending on its concentration.^1^ Strontium‐based therapies – such as the administration of strontium ranelate – have been shown to reduce fracture incidence in osteoporotic patients.^2^, ^3^

Accurate quantification of bone strontium concentrations is important for ensuring that strontium‐based treatments remain within safe and effective limits. Typical baseline concentrations of strontium in human bone range from 50–200 ppm, with higher values observed in patients undergoing strontium‐based therapies. Clinically, quantification at or below 100 ppm is important for monitoring both background exposure and therapeutic uptake. Accurate quantification is also required in order to perform general population studies to further understand strontium's role in bone health. Additionally, precise estimation of bone strontium concentrations is critical when interpreting bone mineral density (BMD) measurements by dual‐energy x‐ray absorptiometry (DXA), as strontium's higher atomic number leads to increased x‐ray attenuation compared to calcium. This results in artificially elevated BMD values in patients undergoing strontium therapy,^4^, ^5^, ^6^, ^7^, ^8^ which can confound clinical management. For this reason, strontium's effect on BMD determinations using quantitative ultrasound has been investigated, yet this method is not currently a clinical standard.^9^, ^10^ Methods of correcting DXA‐determined BMD for bone strontium concentration are still relevant and required in the clinical environment today.

In vivo x‐ray fluorescence (XRF) spectrometry is a promising non‐invasive method for quantifying bone strontium concentrations. It delivers a low radiation dose (∼80 nSv per measurement) and provides detection limits suitable for both healthy individuals and those with elevated strontium levels.^11^, ^12^ The technique enables repeated measurements over time without the risks associated with biopsy. However, its broader clinical adoption is constrained by challenges related to calibration, variability in soft tissue thickness and bone composition, and the absence of standardized protocols.^13^, ^14^ Advancing robust correction methods and standardization is essential to improve the accuracy and reproducibility of in vivo XRF. Moreover, XRF is typically implemented as a stand‐alone system, not easily integrated with standard BMD measurement platforms such as DXA, which limits its ability to provide concurrent assessment of bone density and strontium content in a single integrated scan. Current measurements also take on the order of half an hour to perform,^15^, ^16^ which is not feasible for routine clinical assessment of bone strontium concentrations in the context of BMD determinations.

To address these limitations, imaging systems that intrinsically differentiate between calcium and strontium are needed. The only established framework for correcting BMD in the presence of strontium – the method of Blake and Fogelman^5^ – relies on indirect estimates of bone strontium content based on iliac crest biopsies, animal‐derived scaling factors, and plasma strontium pharmacokinetics. This introduces uncertainty due to both inter‐individual variability and the need for extrapolation from the pelvis to the spine and hip.

Here, we propose a triple‐energy x‐ray imaging technique capable of simultaneously quantifying calcium and strontium concentrations in hand bones, in particular the fingers. Prior studies have shown that hand BMD correlates strongly with BMD at central skeletal sites, including the spine and hip.^17^, ^18^, ^19^ In addition to BMD, projection images of the hand enable assessment of trabecular bone texture, which has emerged as a meaningful marker of bone quality, distinguishing between healthy and osteoarthritic joints.^20^, ^21^ When combined with elemental decomposition, the ability to extract both structural and compositional information from a single low‐dose acquisition positions triple‐energy radiography as a promising approach for comprehensive assessment of bone health at the hand.

Photon‐counting x‐ray detectors (PCDs) provide a powerful platform for triple‐energy imaging by directly converting individual x‐ray photons into electrical signals and sorting them into multiple energy bins in real time.^22^, ^23^, ^24^ This enables basis material decomposition^25^ from a single acquisition, without the need for multiple exposures or beam switching. The strontium K‐edge at ∼16 keV introduces a distinct spectral feature that facilitates separation of strontium from calcium‐rich bone and soft tissue, particularly in the low‐energy range. Recent advances in detector design – including low‐noise electronics and charge summing circuitry^26^, ^27^ – have improved energy resolution and reduced charge‐sharing artifacts, making PCDs well suited for high‐resolution applications such as finger radiography.

In this work, we conduct a simulation study to evaluate the feasibility of the proposed approach by estimating the limits of quantification (LOQs) and corresponding minimum absorbed doses required for strontium concentration measurements in the finger using triple‐energy photon‐counting radiography.

## 2 METHODS

**FIGURE 1:**
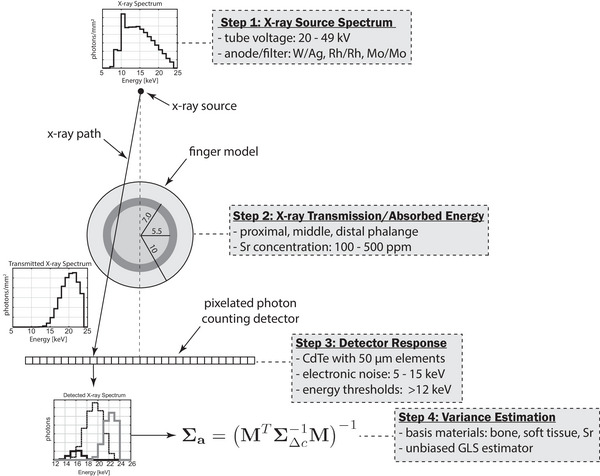
Overview of the simulation pipeline used to evaluate strontium quantification performance. The model includes spectrum generation, transmission through a finger phantom, photon‐counting detector response with noise, and generalized least‐squares estimation of material composition.

An overview of the simulation framework is shown in Figure 1. The goal of the simulation was to determine the LOQ and minimum absorbed dose (D) required for estimating the strontium‐to‐bone concentration ratio using triple‐energy photon‐counting x‐ray imaging. The model incorporates realistic x‐ray spectra, an anatomical representation of the human finger, and a pixelated CdTe‐based photon‐counting detector with energy thresholding. A generalized least‐squares (GLS) estimator was assumed to be unbiased, and the corresponding variance in the estimated strontium‐to‐bone ratio was computed from the measurement model and noise covariance. The analysis was repeated across multiple anode/filter combinations, tube voltages, energy thresholds, and electronic noise levels to identify conditions that minimize the LOQ. Details of each component are provided in the following subsections.

### 2.1 Triple‐energy imaging

The number of photons counted in an energy bin of a photon‐counting detector was modeled as

(1) $$c_{i}=\int_{0}^{\infty }\phi_{0}\left(E\right)G_{i}\left(E\right)e^{-\mu_{\text{Sr}}a_{\text{Sr}}-\mu_{B}a_{B}-\mu_{\text{ST}}a_{\text{ST}}}dE,\;\;i=1..3$$

where $\phi_{0}\left(E\right)$ (${\mathrm{mm}}^{-2}\mathrm{keV}$ 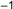) represents the unattenuated, energy‐dependent fluence incident on the detector, the a’s (${g/\mathrm{cm}}^{2}$) represent the areal densities of strontium, bone (B) and soft‐tissue (ST), $G_{i}\left(E\right)$ (${\mathrm{mm}}^{2}$) represents the large‐area gain of energy bin i,^28^ and μ represents the mass attenuation coefficient, which is energy‐dependent. The large‐area gain $G_{i}\left(E\right)$ is the number of photons counted in energy bin i per unit incident energy at energy E per unit fluence at energy E, that is, $G_{i}\left(E\right)=\left(dc_{i}/dE\right)\left(1/\phi \left(E\right)\right)$. It incorporates detector physics such as charge cloud effects, electronic noise, and energy thresholding. The term “large‐area” indicates that all detections are counted, regardless of where they occur relative to the site of x‐ray interaction.^28^, ^29^

Note that scatter was omitted from the model in Equation (1). This simplification is justified for several reasons. First, we focused on low tube voltages (<50 kVp) due to the location of the strontium K‐edge (∼16 keV). In this energy range, the small size of the finger and the dominance of photoelectric interactions, particularly in bone, result in a relatively low scatter fraction. For example, at energies up to 25 keV, over 80% of x‐ray interactions in bone are photoelectric. Although this fraction drops to approximately 50% in soft tissue, overall attenuation by soft tissue is limited – less than 20% for energies above 25 keV – due to the short path lengths involved. Second, the model assumes an air gap between the object and detector, which further mitigates scatter by allowing off‐axis photons to miss the detector.

For bone‐strontium estimation, the objective is to invert the system of equations defined by Equation (1) to recover the unknown areal densities of the three materials. From these, the strontium‐to‐bone concentration by mass can be computed as:

(2) $$C=\frac{a_{\text{Sr}}}{a_{B}}.$$

### 2.2 Generalized least‐squares estimation of areal densities

We assume $a_{\mathrm{Sr}}$, $a_{B}$, and $a_{\mathrm{ST}}$ are estimated using an unbiased generalized least‐squares (GLS) estimator, which provides a theoretical lower bound on the variance of the estimates. This approach establishes a benchmark for the best achievable performance, enabling quantitative comparisons with both experimental results and novel estimators.

To formulate the problem, we linearize the system about the local background, yielding:

(3) $$c_{i}\approx c_{i,0}\left(1-\sum_{j=1}^{N}M_{i,j}\Delta a_{j}\right)$$

where $c_{i,0}$ is the expected count in bin i in the local background, $\Delta a_{j}$ is the perturbation in areal density of material j, and $M_{i,j}$ is an effective attenuation coefficient of material j for energy bin i:

(4) $$M_{i,j}=\frac{\int_{0}^{\infty }\phi_{0}\left(E\right)G_{i}\left(E\right)T_{0}\left(E\right)\mu_{j}\left(E\right)dE}{\int_{0}^{\infty }\phi_{0}\left(E\right)G_{i}\left(E\right)T_{0}\left(E\right)dE}.$$

where $T_{0}\left(E\right)$ represents the transmission fraction through the local background.

The perturbation in normalized counts, $\left(c_{i}-c_{i,0}\right)/c_{i,0}$, can then be expressed as:

(5) $$\Delta \tilde{c}=-M\Delta a+\varepsilon .$$

where ε represents measurement noise with covariance matrix $\Sigma_{\Delta c}$. In this work we assume a system that implements analog charge summing, for which energy‐bin counts are approximately Poisson distributed, the off‐diagonal elements of $\Sigma_{\Delta c}$ are negligible, and the noise can be treated as uncorrelated across bins.^30^, ^31^ In this case, $\Sigma_{\Delta c}$ is a diagonal matrix with diagonal elements given approximately by $c_{i}^{-1}$. Under the GLS framework, the covariance matrix of the estimated areal densities is given by:

(6) $$\Sigma_{a}={\left(M^{T}\Sigma_{\Delta c}^{-1}M\right)}^{-1}.$$

### 2.3 Limit of quantification

The goal of triple‐energy imaging in this work is not to produce an image, but to estimate the bone‐strontium concentration. We therefore assume an approach that averages the bone‐strontium concentration over a single finger. Our finger model is described in Section 2.4. Letting N represent the number of image pixels used to compute the average, the bone strontium concentration is estimated as

(7) $$\langle C\rangle =\langle \frac{a_{\mathrm{Sr}}}{a_{B}}\rangle =\frac{1}{N}\sum_{n=1}^{N}\frac{a_{\mathrm{Sr},n}}{a_{B,n}}$$

where $a_{\mathrm{Sr},n}$ and $a_{B,n}$ represent the areal densities of strontium and bone for pixel n. Our goal is to estimate the SNR of Equation (7) with the goal of estimating limits of detection. We assume a detector that implements analog charge summing, for which estimates of areal densities from different pixels are independent of one another. The estimates of $a_{\mathrm{Sr},n}$ and $a_{B,n}$ from the same pixel are, however, correlated, as described by Equation (6). Accounting for these correlations yields the following expression for the variance of Equation (7):

(8) $$\sigma_{\langle C\rangle }^{2}=\frac{1}{N^{2}}\sum_{n=1}^{N}{\left(\frac{a_{\mathrm{Sr},n}}{a_{B,n}}\right)}^{2}\left(\frac{\sigma_{\mathrm{Sr},n}^{2}}{a_{\mathrm{Sr},n}^{2}}+\frac{\sigma_{B,n}^{2}}{a_{B,n}^{2}}-2\frac{\sigma_{\mathrm{Sr},B,n}^{2}}{a_{\mathrm{Sr},n}a_{B,n}}\right)$$

where $\sigma_{\mathrm{Sr},n}^{2}$ and $\sigma_{B,n}^{2}$ are the diagonal elements of $\Sigma_{\Delta a}$ calculated using Equation (6), and $\sigma_{\mathrm{Sr},B,n}^{2}$ represents the covariance between the strontium and bone areal densities, calculated from the off‐diagonal elements of $\Sigma_{\Delta a}$. Since areal densities of both strontium and bone vary as a function of position within the image, they are both functions of n. Additionally, since the noise in the image is largely determined by the number of photons reaching the detector, the variances are also functions of the index n.

We define the LOQ as the concentration corresponding to ten times the standard deviation of the measured signal from a sample containing no strontium:

(9) $$\mathrm{LOQ}=10\sigma_{{\langle C\rangle }_{b}}$$

where the subscript b indicates a “blank” sample. The factor of 10 in Equation (9) follows the conventional IUPAC definition of the LOQ, defined as ten times the standard deviation of the blank sample.^32^ This threshold ensures a sufficiently high confidence in distinguishing signal from background fluctuations.

Since the variance scales inversely with the absorbed radiation dose (D) (Gy), we can compute the minimum required dose to achieve a desired LOQ as:

(10) $$D_{\min}=\frac{100{\tilde{\sigma }}_{{\langle C\rangle }_{b}}^{2}}{{\mathrm{LOQ}}^{2}}$$

where ${\tilde{\sigma }}_{{\langle C\rangle }_{b}}^{2}=\sigma_{{\langle C\rangle }_{b}}^{2}D_{b}$ (Gy) where $D_{b}$ is the absorbed dose corresponding to $\sigma_{{\langle C\rangle }_{b}}^{2}$; the quantity ${\tilde{\sigma }}_{{\langle C\rangle }_{b}}^{2}$ is dose‐independent.

### 2.4 Finger model

**FIGURE 2:**
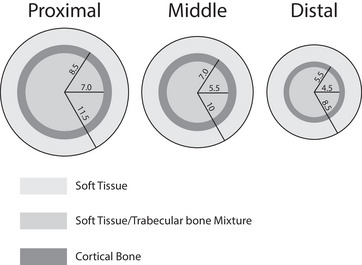
Cross‐sectional schematics of the proximal, middle, and distal phalanges of the middle finger, showing the modeled layers of soft tissue, cortical bone, and trabecular/soft tissue mixture. Radii are labeled in millimeters.

We modeled the three phalanges of the middle finger, representing each as a cylindrical shell of cortical bone surrounding an inner core modeled as a homogeneous mixture of trabecular bone and soft tissue. Additionally, a 3 mm thick layer of soft tissue – representing skin, connective tissue, and muscle – was included surrounding the cortical bone. A cross‐section of each phalanx model is shown in Figure 2, which also indicates the diameters of the cortical shell and trabecular core. The lengths of the proximal, middle, and distal phalanges were taken to be 30, 25, and 20 mm, respectively.

The mass density of cortical bone was taken as 1.65 ${g/\mathrm{cm}}^{3}$. The trabecular core was assumed to have a porosity (p) of 0.8,^33^, ^34^ yielding an effective mass density of 0.33 ${g/\mathrm{cm}}^{3}$ (=0.2 × 1.65 ${g/\mathrm{cm}}^{3}$). Attenuation due to soft tissue within the trabecular core was modeled as water with a density of 0.8 ${g/\mathrm{cm}}^{3}$ (=0.8 × 1.0 ${g/\mathrm{cm}}^{3}$).

For a given strontium concentration (C), the mass densities of strontium and bone in the resulting mixture were calculated using:

(11) $${\tilde{\rho }}_{\mathrm{Sr}}=C{\left(\frac{C}{\rho_{\mathrm{Sr}}}+\frac{1-C}{\rho_{B}}\right)}^{-1}$$

and

(12) $${\tilde{\rho }}_{B}=\left(1-C\right){\left(\frac{C}{\rho_{\mathrm{Sr}}}+\frac{1-C}{\rho_{B}}\right)}^{-1}$$

where $\rho_{\mathrm{Sr}}$ (=2.64 ${g/\mathrm{cm}}^{3}$) and $\rho_{B}$ (=1.65 ${g/\mathrm{cm}}^{3}$) are the mass densities of pure strontium and bone, respectively. Strontium is known to accumulate at higher concentrations in trabecular bone compared to cortical bone; we assumed a fixed mass concentration ratio of 4:1 between trabecular and cortical bone when assigning strontium concentrations within the model.

**FIGURE 3:**
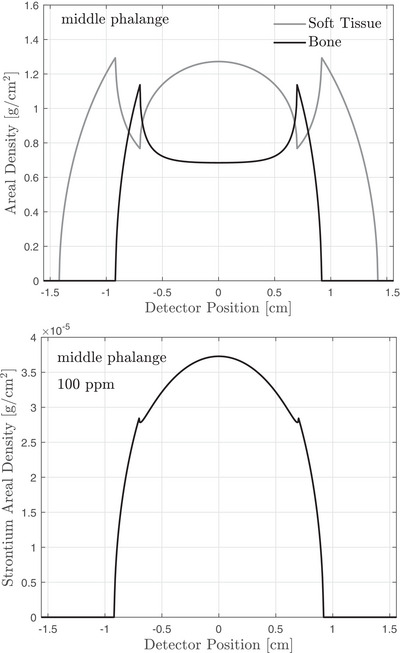
Projected areal densities of bone, soft tissue and strontium for the finger model described in Section 2.4.

Path lengths through cortical bone, trabecular bone, and soft tissue were computed assuming a divergent beam in the fan direction and a parallel beam in the cone direction. These path lengths correspond to rays that bisect each detector element and were calculated using simple geometric considerations. A source‐to‐detector distance (SDD) of 15 cm and a source‐to‐object distance (SOD) of 10 cm were used, where the object distance refers to the center of the finger. Example projected areal densities are shown in Figure 3. This simplified geometric model was used to simulate x‐ray attenuation.

### 2.5 Detector model

The effect of the detector response is captured by the large‐area gain $G_{i}\left(E\right)$ (mm²) of the energy bins. We used a validated model^28^, ^30^ of the energy response of a cadmium telluride (CdTe) PCD to compute the large‐area gain. The model accounts for characteristic x‐ray emission and subsequent escape or reabsorption, charge cloud expansion due to diffusion and Coulomb repulsion, electronic noise, and energy thresholding. It has been calibrated against empirical data, yielding zero‐frequency detective quantum efficiency and SNR predictions that agree reasonably well with experimental results.^30^, ^35^, ^36^, ^37^, ^38^

We investigated the LOQ of strontium concentration estimation as a function of electronic noise level. A pixel pitch of 50 μm was considered, which – though not necessary for strontium estimation – reflects a forward‐looking interest in capturing trabecular micro‐structure in future high‐resolution imaging applications. Importantly, because analog charge summing was assumed, the impact of pixel size on LOQ is modest; the summed energy across a cluster of elements mitigates much of the degradation that would otherwise occur due to charge sharing at small pixel sizes. Charge sharing correction is already implemented in several commercially available ASICs, including those from Medipix and Varex, making this a realistic modeling assumption.^26^, ^27^

Simulations were performed for electronic noise floors of 5, 10, and 15 keV, capturing the spread in performance observed across currently available detector technologies. The CdTe sensor was modeled with a thickness of 750 μm, consistent with our calibration measurements, although this thickness exceeds what is required for the low‐kVp application considered here. Charge sharing correction was implemented using analog charge summing based on the approach described by Ullberg et al.,^27^ in which each pixel communicates with its eight nearest neighbors. When simultaneous pulses are detected in multiple neighboring elements, they are assumed to originate from the same x‐ray interaction. A count is then assigned to the pixel with the largest pulse. In our implementation, for each x‐ray interaction, the total energy deposited in a 3×3 cluster centered on the pixel with the maximum energy was summed, and a count was attributed to that central pixel.

### 2.6 Optimization

We evaluated three x‐ray tube anode/filter combinations: tungsten (W) with 50 μm of silver filtration, rhodium (Rh) with 25 μm of rhodium filtration, and molybdenum (Mo) with 30 μm of molybdenum filtration. Spectrum data were obtained from Boone et al.^39^ These filters were selected to shape the incident spectra appropriately for finger imaging, where bone preferentially attenuates low‐energy photons. We focused on mammography‐like spectra because the K‐edge of strontium lies near 16 keV, suggesting that low‐kVp beams – specifically those below 50 kVp – would be most effective for improving sensitivity to strontium. Accordingly, we limited our analysis to tube voltages between 20 and 50 kVp.

For triple‐energy acquisition, we optimized the selection of tube voltage and energy thresholds that define the low, medium, and high energy bins. The lowest energy threshold, $E_{1}$, was set to 12 keV for noise floors of 5 and 10 keV, and to 15 keV for the 15 keV noise floor. The choice of 12 keV for the 5 and 10 keV noise floors was made to reduce Swank noise arising from double‐counting of low‐energy photons.^30^, ^40^ It was further justified by the fact that x‐ray spectra transmitted through the finger contain negligible photons below 12 keV, due to strong attenuation by bone.

Optimization of tube voltage and energy thresholds was performed using a grid search. For each anode/filter combination and electronic noise level, the algorithm evaluated SNR normalized to absorbed dose over a grid of tube voltages ranging from 20 to 50 kVp in 1 kVp steps. For each voltage, the first threshold $E_{1}$ was fixed based on the electronic noise floor (either 12 or 15 keV). The second threshold $E_{2}$ was varied from $E_{1}+2\;\mathrm{keV}$ up to tube voltage −2 keV. The third threshold $E_{3}$ was varied from $E_{2}+2$ keV up to tube voltage −1 keV. The combination of tube voltage and thresholds that maximized the signal‐to‐noise ratio (SNR) per unit dose was selected as the optimal configuration. This exhaustive approach ensured identification of globally optimal settings under the simulation assumptions.

This optimization was performed across all combinations of anode material, tube voltage, and electronic noise level. The objective function was based on the average areal densities of strontium, bone, and soft tissue. For the optimization, a strontium concentration of 150 ppm was assumed in trabecular bone, reflecting average baseline levels in healthy adult humans.^11^

### 2.7 Absorbed dose estimate

Absorbed dose was estimated using a simplified approach. For each detector pixel, we calculated the energy deposited along the ray path connecting the pixel to the x‐ray source by summing the energy of all photons that interacted along that path. This deposited energy was then multiplied by the pixel area to obtain the total energy per pixel. The mass associated with each pixel was estimated by summing the areal mass densities of the materials (soft tissue, trabecular bone, and cortical bone) along the same ray path and multiplying by the pixel area. The absorbed dose was then computed as the energy deposited divided by the corresponding mass.

### 2.8 Projected Fisher information analysis

To quantify the relative contribution of each energy bin to the precision of the estimated strontium‐to‐bone concentration, we computed the Fisher information projected along the gradient of the strontium/bone ratio. This approach captures how much information each bin provides specifically for estimating the ratio $C=a_{\mathrm{Sr}}/a_{B}$, rather than for general material decomposition.

For each energy bin i, we computed its contribution to the total Fisher information matrix as:

(13) $$I_{i}=\frac{1}{\sigma_{i}^{2}}M_{i}^{T}M_{i}$$

where $M_{i}$ is the ith row of the sensitivity matrix M, and $\sigma_{i}^{2}={\bar{c}}_{i}^{-1}$ is the variance of the residual in that bin. Each $I_{i}$ is a rank‐one matrix that represents the information content of bin i across the three estimated areal densities.

To isolate the information relevant to the strontium/bone ratio, we projected each bin' s Fisher matrix onto the gradient of C with respect to the areal density vector $a={\left[a_{\mathrm{Sr}}\;a_{B}\;a_{\mathrm{ST}}\right]}^{T}$. The gradient vector is:

(14) $$\nabla C={\left[\begin{array}{ccc} \frac{1}{a_{B}}, & -\frac{a_{\mathrm{Sr}}}{a_{B}^{2}}, & 0 \end{array}\right]}^{T}.$$

The projected Fisher information from bin i is then:

(15) $$I_{i}=\nabla C^{T}I_{i}\nabla C.$$

These scalar values represent the contribution of each bin to the estimation precision of C. We normalized the projected contributions across the three bins to obtain fractional contributions.

This analysis was performed at the optimal energy thresholds and tube voltages determined for each anode/filter combination and electronic noise level.

## 3 RESULTS

### 3.1 Optimal technique parameters

Optimization of the imaging parameters identified the most effective combinations of tube voltage and energy thresholds for each anode/filter configuration across varying levels of electronic noise.

**FIGURE 4:**
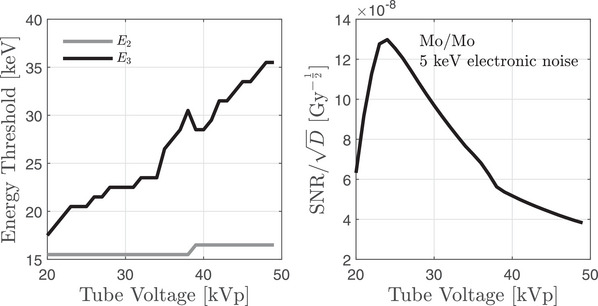
Optimization results for Mo/Mo at 5 keV electronic noise. (Left) Optimal thresholds $E_{2}$ and $E_{3}$ versus tube voltage. (Right) SNR normalized to absorbed dose.

Figure 4 shows an example of the optimization over tube voltage and energy thresholds for the Mo/Mo anode/filter combination at 5 keV electronic noise. The left panel illustrates how the optimal thresholds $E_{2}$ and $E_{3}$ vary with tube voltage. The lower threshold $E_{2}$ remains just below the strontium K‐edge, while the upper threshold $E_{3}$ increases steadily with tube voltage, likely to increase spectral separation. The right panel shows the corresponding signal‐to‐noise ratio normalized to dose, which peaks near 25 kVp – where the spectrum provides sufficient photon flux just above and below the strontium K‐edge – and declines at higher voltages due to reduced flux near the K‐edge for matched dose.

**FIGURE 5:**
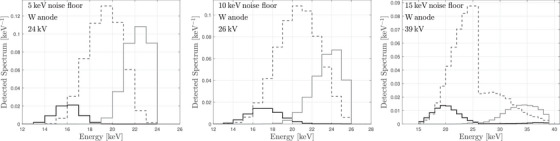
Spectra seen by each energy bin at the optimal thresholds and tube voltages for a tungsten anode.

Trends in optimal voltage and thresholds are illustrated more broadly in Table 1, which summarizes results across multiple anode/filter combinations and electronic noise floors. For detectors with a 5 keV noise floor, the optimal tube voltages were 24–27 kVp, with thresholds of 12.5, 15.5, and 20.5–21.5 keV depending on the anode. Increasing the noise floor to 10 keV resulted in slightly higher optimal voltages (24–28 kVp) and raised the highest energy threshold to 22.5 keV. At a 15 keV noise level, optimal voltages increased substantially (up to 49 kVp for Rh/Rh and Mo/Mo targets), with corresponding energy thresholds also shifting upward to 15, 19, and 31–36 keV. Figure 5 illustrates the spectral separation achieved at these optimized settings using a W/Ag spectrum. The curves in this figure represent the product of the incident x‐ray spectrum (including added filtration), the transmission fraction through a representative ray path in the finger, and the large‐area gain of the detector, which accounts for x‐ray interaction physics, electronic noise, and energy thresholding. The discontinuity near 26 keV in the mid‐energy bin at 39 kVp is due to the silver filter, which introduces a sharp reduction in photon fluence at its K‐edge (∼26 keV). Also noteworthy is that analog charge summing has mostly reduced the sensitivity of the low‐energy bins to high‐energy photons. The figure confirms that the selected thresholds effectively divide the transmitted x‐ray spectrum into three distinct regions, each tailored to balance strontium sensitivity and noise under realistic detector constraints.

**TABLE 1:** Optimal tube voltages and energy thresholds as a function of target/filter combination and electronic noise level.

	5 keV noise floor					10 keV noise floor					15 keV noise floor				
Target/Filter	Tube voltage	$E_{1}$	$E_{2}$	$E_{3}$	$D/K_{\mathrm{air}}$ *	Tube voltage	$E_{1}$	$E_{2}$	$E_{3}$	$D/K_{\mathrm{air}}$	Tube Voltage	$E_{1}$	$E_{2}$	$E_{3}$	$D/K_{\mathrm{air}}$
W/Ag	24	12.5	15.5	20.5	1.07	26	12.5	15.5	22.5	1.20	39	15.0	18.0	31.0	1.71
Rh/Rh	27	12.5	15.5	21.5	0.69	28	12.5	15.5	22.5	0.74	49	15.0	19.0	36.0	1.57
Mo/Mo	24	12.5	15.5	20.5	0.85	24	12.5	15.5	21.5	0.85	49	15.0	19.0	35.0	1.85

* Represents absorbed dose [μGy] per unit air kerma [mGy].

### 3.2 Limits of quantification and absorbed dose

**FIGURE 6:**
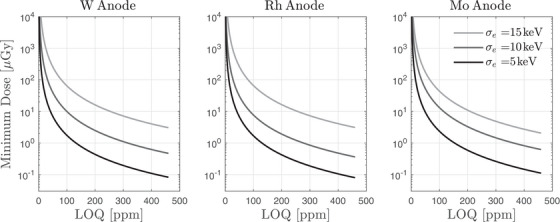
Minimum dose as a function of LOQ as a function of anode and electronic noise level.

Figure 6 shows the relationship between the minimum absorbed dose and the LOQ for strontium concentration, plotted for each anode/filter combination across three levels of electronic noise. As expected, the required dose increases sharply as the desired LOQ decreases, reflecting the inverse‐square dependence of dose on measurement precision. Electronic noise had a substantial effect: lower noise floors consistently enabled lower dose requirements for a given LOQ, emphasizing the importance of low‐noise detector electronics. For example, achieving an LOQ of 100 ppm required only ∼13 μGy at a 5 keV noise floor, compared to ∼100 μGy at 10 keV and over 175 μGy at 15 keV. In contrast, the differences between anode/filter combinations were minimal when each was properly optimized with respect to tube voltage and energy thresholds. This indicates that while spectral shaping does contribute to performance, its impact is modest provided that acquisition parameters are appropriately tuned. These results highlight that detector noise characteristics are the dominant factor in enabling low‐dose quantification of strontium in bone.

### 3.3 Signal‐to‐noise ratio

**FIGURE 7:**
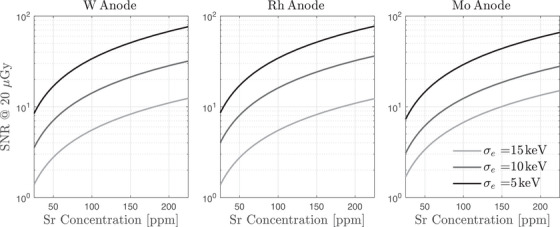
SNR as a function of strontium concentration at 20 μGy absorbed dose.

To further assess the system's sensitivity, Figure 7 presents the signal‐to‐noise ratio of the strontium concentration estimate as a function of true strontium concentration at a fixed absorbed dose of 20 μGy. As expected, SNR increased monotonically with strontium concentration, demonstrating improved quantification performance for higher strontium levels. Importantly, even at relatively low concentrations near 50 ppm, the SNR exceeded unity for detectors with 5 and 10 keV noise floors, indicating the feasibility of detection under clinically relevant conditions. Performance degraded substantially at a 15 keV noise floor, underscoring again the critical role of electronic noise in low‐dose applications. These results suggest that triple‐energy acquisition can enable reliable strontium quantification across a wide dynamic range, provided that detector noise is sufficiently low and exposure conditions are optimized.

### 3.4 Contribution of each energy bin

**FIGURE 8:**
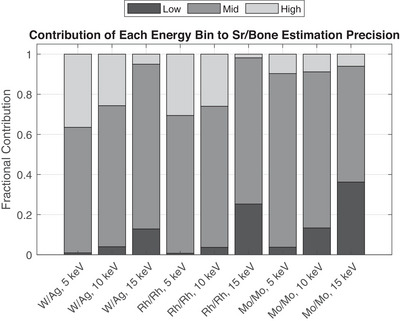
Contribution of each energy bin to the strontium/bone estimation precision.

To evaluate how each energy bin contributes to the precision of the strontium‐to‐bone concentration estimate, we computed the projected Fisher information from each bin along the gradient of the strontium/bone ratio. Figure 8 shows the fractional contribution of each bin to this precision for all combinations of anode/filter spectra and electronic noise levels. The mid‐energy bin consistently contributed the most across all conditions, indicating that it captured the spectral features most relevant for distinguishing strontium from bone. At low electronic noise levels, the high‐energy bin was typically the second most informative, consistent with its sensitivity to higher‐density bone content. As electronic noise increased, the contribution of the low‐energy bin rose and eventually surpassed that of the high‐energy bin. This trend can be explained by the shifting placement of energy thresholds: at low noise levels, the threshold defining the mid bin lies just below the strontium K‐edge (∼16 keV), placing the K‐edge in bin 2. At high noise levels, the optimal thresholds increase, and the K‐edge falls into the low‐energy bin instead. This shift enhances the utility of the low‐energy bin for strontium estimation at higher noise levels, while the high‐energy bin becomes increasingly limited by low photon counts. These findings highlight how the relative information content of each bin depends not only on the spectrum and detector noise, but also on the alignment of energy thresholds with spectral features of the target material.

## 4 DISCUSSION

This simulation study demonstrates the feasibility of using triple‐energy photon‐counting x‐ray imaging for low‐dose quantification of strontium in bone. By modeling finger geometry, x‐ray spectra, and detector response, we estimated LOQs and minimum absorbed doses required to resolve clinically relevant strontium concentrations – establishing a foundation for future experimental validation.

A key finding is that LOQs below 100 ppm are achievable at absorbed doses under 20 μGy, assuming a low electronic noise floor (∼5 keV). This level of sensitivity would enable frequent, non‐invasive monitoring of strontium uptake, sufficient to track baseline levels and therapeutic responses. At higher noise levels, dose requirements rise sharply, underscoring the importance of continued development of low‐noise photon‐counting electronics.

The results also clarify the role of acquisition parameters. While tube voltage and threshold optimization improve performance, the influence of anode/filter selection was modest once other parameters were tuned – suggesting that dose efficiency gains can be realized across a range of systems provided spectral flexibility is available. The mid‐energy bin consistently contributed most to the Fisher information, emphasizing the importance of aligning thresholds with the strontium K‐edge (∼16 keV).

Although our primary focus was compositional estimation, the projection‐based approach preserves spatial resolution and supports additional analyses such as trabecular texture assessment. Previous work has shown that hand radiographs capture micro‐architectural information relevant to bone quality, particularly in osteoarthritic populations. Integrating elemental and structural measurements into a single scan could support more comprehensive bone health assessments. From a translational standpoint, this method could be implemented in compact peripheral imaging systems tailored to the hand or wrist, offering a practical and low‐risk tool for monitoring strontium uptake. Such systems could complement DXA or serve as standalone modalities in settings where traditional densitometry is impractical.

It is worth noting that no commercially available photon‐counting detector currently integrates all three capabilities required for this application – low electronic noise, three independently configurable energy bins, and hardware‐based charge sharing correction. Each of these features is available individually in existing systems, but not yet in combination. The absence of hardware‐based charge sharing correction has a measurable impact on quantification performance. Recent work by Aubert et al.^41^ demonstrated that omitting this correction results in approximately a twofold degradation in SNR for iodine imaging, and Dahri et al.^35^ reported similar findings in the context of thoracic imaging. A twofold reduction in SNR can be recovered by increasing the dose fourfold. Given the already low baseline dose of the proposed method, this tradeoff may be acceptable. Separately, the absence of three configurable energy bins restricts true triple‐energy imaging. A workaround is to acquire two exposures with different energy thresholds – a strategy that effectively doubles the dose. In the context of finger imaging, where the baseline dose is already low, this increase may be acceptable. The combination of both limitations – lack of charge sharing correction and the need for multiple exposures – has compounding effects: reduced SNR due to charge sharing, and increased dose due to repeated exposures. Together, these could undermine the low‐dose, high‐precision goals of the proposed technique. As such, the development of next‐generation detectors that combine low noise, multi‐bin configurability, and integrated charge sharing correction will be key to translating this method into clinical practice.

Our modeling approach includes several limitations. While some detector non‐idealities – such as fluorescence escape and charge sharing – were explicitly modeled using experimentally validated approaches, other effects were omitted. In particular, x‐ray scatter and pulse pile‐up were not simulated. Scatter is expected to be minimal for finger imaging due to the small volume and narrow beam path, and recent work by Aubert et al.^42^ has shown that optimal threshold selection for k‐edge imaging is relatively insensitive to moderate scatter levels. Regarding pile‐up, the expected count rates in this low‐dose application are well within the linear operating range of commercial flat‐panel photon‐counting detectors, such as that characterized by Dahri et al.,^35^ minimizing concern for pile‐up artifacts.

The anatomical model in this work used piecewise‐constant concentric layers to approximate the geometry of the finger. While this approach is suitable for first‐order performance assessment, it does not capture the continuous variation in bone density – that is, gradual spatial changes in mineral content – found in both cortical and trabecular bone. However, the estimator used in this study computes an average strontium‐to‐bone concentration across the full cross‐section of the finger, which mitigates the influence of local anatomical variability and supports the use of spatially simplified models for evaluating system‐level performance. Future studies will incorporate more anatomically realistic models to assess the robustness of the estimator under spatially varying conditions.

Absorbed dose was estimated using a first‐order approach based on path‐integrated energy deposition. This method assumes complete local energy deposition for all photon interactions and does not account for scatter out of the volume, fluorescence escape, or dose contributions from scattered photons originating outside the beam path. As a result, it likely overestimates total absorbed dose and, by extension, the minimum doses required to achieve a given LOQ. Nevertheless, it offers a simple and conservative metric for comparing imaging protocols or acquisition parameters.

Pixel‐to‐pixel variations in the detector' s energy response, if uncorrected, can bias material decomposition and degrade LOQ performance. In this theoretical study, we assumed a uniform large‐area gain $G_{i}\left(E\right)$ to isolate the performance limits of the proposed method. In experimental systems, such variations are typically addressed through pixel‐wise spectral calibration, which has been shown to improve spectral accuracy and reduce ring artifacts in photon‐counting CT.^43^ Adopting such calibration approaches will be essential in translating these theoretical limits into practical measurement capabilities.

Scatter was not explicitly modeled in this study. Although scatter is expected to be minimal due to the small object size, low tube voltage, and presence of an air gap, even modest scatter contributions may degrade material decomposition. In clinical systems, strontium estimation would likely rely on empirical calibration under realistic scatter conditions, allowing scatter effects to be implicitly incorporated into the estimator. One specific concern is the impact of scatter on the noise distribution: recent work has shown that scatter tends to accumulate disproportionately in low‐energy bins,^44^ which may bias or reduce the precision of K‐edge–based estimates if not properly accounted for.^45^ The key challenge is ensuring estimator robustness when clinical conditions differ from those used during calibration. Although empirical estimators could, in principle, be investigated in simulation, the primary objective of this work was to establish theoretical performance limits based on an idealized model. These limits provide valuable benchmarks for future empirical methods.

Pulse pileup was not modeled, as count rates in our simulated exposures remain well within the linear operating range (<10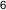 counts/${\mathrm{mm}}^{-2}$/$s^{-1}$) of commercially available flat panel PCDs.^35^ Under these conditions, pileup‐induced spectral distortion is expected to be minimal.

In view of these limitations, the LOQs reported in this work represent theoretical lower bounds under ideal conditions, assuming an accurate forward model and noise characterization. These bounds were computed using a GLS estimator, which achieves minimum variance when the model is correctly specified. While real‐world systems may include effects not modeled here – such as scatter, pileup, or pixel‐to‐pixel detector variability – these are expected to be modest in the imaging regime considered. The results should therefore be interpreted as theoretical benchmarks that can guide the development and calibration of empirical estimators. Notably, empirical methods that do not require explicit knowledge of the forward model have been shown to achieve the Cramér–Rao lower bound in K‐edge imaging applications.^46^, ^47^ Future work will incorporate robustness testing, empirical estimator development, and experimental validation under clinically relevant conditions.

## 5 CONCLUSIONS

This study demonstrates the feasibility of using triple‐energy photon‐counting x‐ray imaging for low‐dose quantification of strontium in bone. Using simulations based on finger geometry and an unbiased generalized least‐squares estimator, we quantified the theoretical minimum absorbed dose required to achieve target levels of precision. Specifically, we assessed the LOQ across a range of acquisition settings and electronic noise levels. At low electronic noise floors (i.e., ∼5 keV), an LOQ of 100 ppm could be achieved with absorbed doses on the order of 20 μGy, substantially lower than those used in standard diagnostic radiography. Higher noise levels required higher doses to maintain the same level of precision. Anode/filter selection had only a modest effect when acquisition parameters were optimized, highlighting detector noise as the dominant factor influencing system performance.

These results provide quantitative benchmarks for the design and optimization of photon‐counting systems for bone strontium measurement. They underscore the importance of minimizing electronic noise and selecting energy thresholds that maximize sensitivity to strontium. The findings support the feasibility of compact, low‐dose imaging systems for noninvasive bone strontium quantification.

## CONFLICT OF INTEREST STATEMENT

The authors declare no conflicts of interest.
